# Numerical Equivalence of Diabatic and Adiabatic Representations in Diatomic Molecules

**DOI:** 10.1021/acs.jctc.3c01150

**Published:** 2024-01-03

**Authors:** Ryan P. Brady, Charlie Drury, Sergei N. Yurchenko, Jonathan Tennyson

**Affiliations:** Department of Physics and Astronomy, University College London, Gower Street, London WC1E 6BT, U.K.

## Abstract

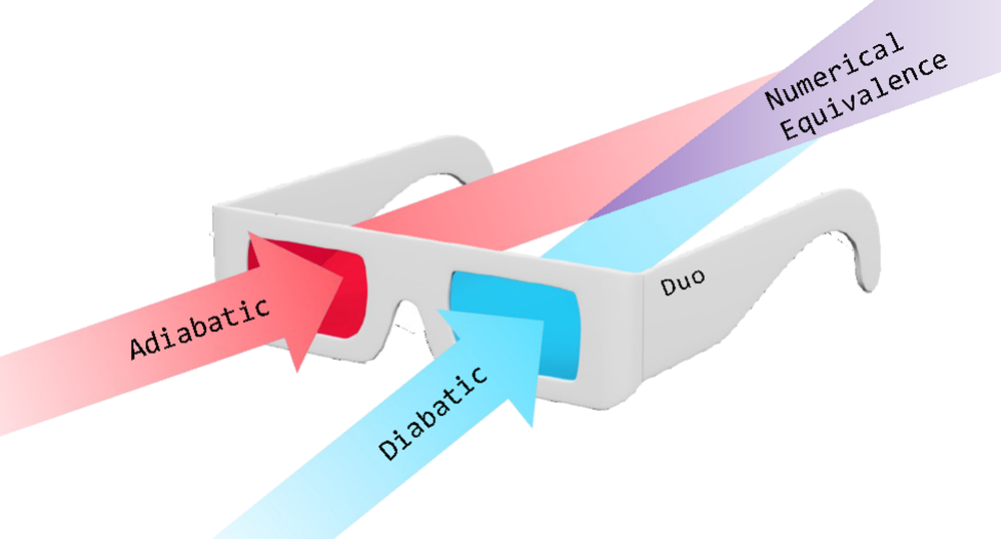

The (time-independent) Schrödinger equation for atomistic systems is solved by using the adiabatic potential energy curves (PECs) and the associated adiabatic approximation. In cases where interactions between electronic states become important, the associated nonadiabatic effects are taken into account via derivative couplings (DDRs), also known as nonadiabatic couplings (NACs). For diatomic molecules, the corresponding PECs in the adiabatic representation are characterized by avoided crossings. The alternative to the adiabatic approach is the diabatic representation obtained via a unitary transformation of the adiabatic states by minimizing the DDRs. For diatomics, the diabatic representation has zero DDR and nondiagonal diabatic couplings ensue. The two representations are fully equivalent and so should be the rovibronic energies and wave functions, which result from the solution of the corresponding Schrödinger equations. We demonstrate (for the first time) the numerical equivalence between the adiabatic and diabatic rovibronic calculations of diatomic molecules using the *ab initio* curves of yttrium oxide (YO) and carbon monohydride (CH) as examples of two-state systems, where YO is characterized by a strong NAC, while CH has a strong diabatic coupling. Rovibronic energies and wave functions are computed using a new diabatic module implemented in the variational rovibronic code Duo. We show that it is important to include both the diagonal Born–Oppenheimer correction and nondiagonal DDRs. We also show that the convergence of the vibronic energy calculations can strongly depend on the representation of nuclear motion used and that no one representation is best in all cases.

## 1 Introduction

Nonadiabatic effects within the electronic structure of molecules are important for many physical and chemical processes^1−7^ such as when a chemical reaction alters the electronic structure, affecting nuclear dynamics. Nonadiabatic processes are also important in astronomy and atmospheric chemistry, where collisions of free radicals and open-shell molecules with spatially degenerate electronic states are often seen.^8−12^ Modeling electronically nonadiabatic processes has also been effective in explaining the bonding in dications such as BF^2+^^13^ and strongly ionic molecules such as LiF^14^ and NaCl,^15^ whose ^1^Σ^+^ ground states show nonadiabatic behavior.

**Figure 1:**
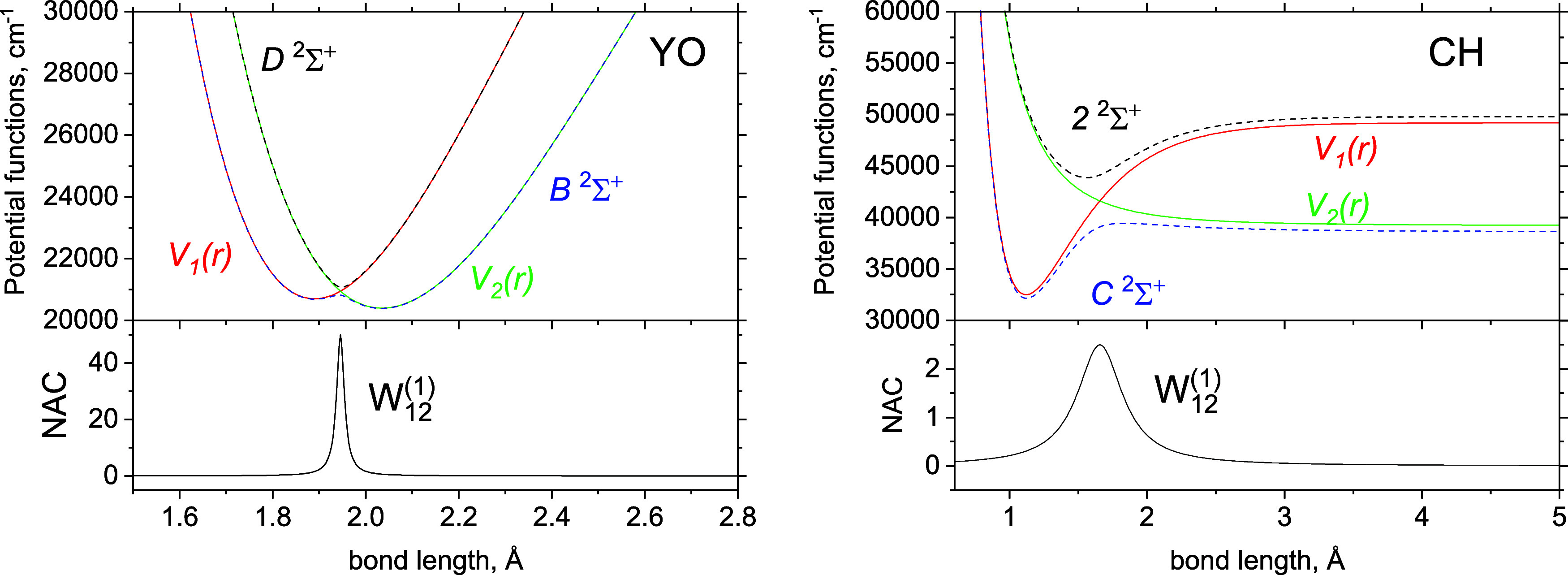
Illustration of the [*D*^2^Σ^+^, *B*^2^Σ^+^] and [C ^2^Σ^+^, 2 ^2^Σ^+^] avoided crossing systems ([black, blue] lines) for the YO and CH diatomic molecules, respectively, which we use to perform tests on the adiabatic and diabatic equivalence. The top panels show the adiabats (solid lines) and diabats (dashed lines). The bottom panels show the corresponding NAC and DC (in units of Å and cm^–1^) of the transformations.

Both the adiabatic and Born–Oppenheimer (BO) approximations assume nuclear dynamics evolve on single electronic potential energy surfaces (PESs),^8^ where no kinetic energy coupling (DDR) to neighboring electronic states occurs and is generally good for predicting near-equilibrium properties for many molecules.^6^ While related, the adiabatic approximation differs from the BO approximation by the addition of the well-known diagonal BO correction (DBOC), introducing mass dependence into the PECs within the adiabatic representation. The adiabatic approximation then breaks down when electronic states of the same symmetry near spatial degeneracy exhibit an avoided crossing. Neumann and Wigner^16^ formalized this as a noncrossing rule for diatomics, showing that potential energy curves (PECs) cannot cross and appear to “repel” upon approach (see Figure 1 for example). Relaxation of the BO and adiabatic approximation is then required to fully encounter the electronically nonadiabatic effects because of the inherent coupling between electronic and nuclear degrees of freedom for both the diagonal and nondiagonal terms.

The so-called derivative couplings (DDRs) or nonadiabatic couplings (NACs) between states that exhibit avoided crossings arise through the nuclear kinetic energy operator acting on the electronic wave functions when the BO approximation is relaxed and corresponds to derivatives in terms of the nuclear coordinate. The computation of DDRs and PESs around the avoided crossing geometry is a major source of computational expense within both quantum chemistry and nuclear motion calculations because of the cusp-like behavior of the PESs and the singular nature of the DDRs at the geometry of spatial degeneracy.^8,17−19^ It is therefore the main focus of many works to explore property-based diabatization methods^8,20−22^ that transform to a diabatic representation, where DDRs vanish or are reduced and PESs become smooth. For diatomics, the smoothness condition of their PECs uniquely defines the unitary transformation to the diabatic representation where NACs (first-order nondiagonal DDR) vanish, PECs are allowed to cross, and consequently, the molecular properties are smooth, at the cost of introducing off-diagonal diabatic potential couplings. This smoothness is then favorable for nuclear motion calculations since no quantities within the molecular model are singular/cusped, making their integration and fitting of analytical forms much simpler. The other method of diabatization, known as point-diabatization,^14,22−30^ is direct and requires the NAC to be obtained *ab initio* such as through the DDR procedure,^31^ where each point can be diabatized without knowledge of the previous one, unlike property-based methods.

Mead and Truhlar^32^ showed that a strictly diabatic electronic basis, in which all derivative coupling vanishes, can be defined for a diatomic system. The conditions required to make the first-order NAC vanish are straightforward; however, a true diabatic electronic basis only exists when one can remove the second-order (diagonal) derivative coupling simultaneously, which is only possible when considering an isolated two-state system, allowing one to ignore coupling to other adiabatic states. The adiabatic to diabatic transformation (AtDT) for the *N*-nuclear-coordinate case up to coupled 4-state systems has been investigated thoroughly by Baer and coauthors since the late 1980s.^33−37^ These works develop the so-called line-integral approach in solution to the matrix differential equation that arises when solving for the AtDT, which completely reduces the NAC matrix. Their results, albeit from a different angle than in this study, are consistent with the results we present.

Despite diabatization being used routinely to treat the avoided crossings of molecular PESs, there have been very few studies examining the numerical equivalence of adiabatic and diabatic states. This would be of value not only to those who want to benchmark their own nuclear motion codes but also to better understand the roles of each term in the diabatic and adiabatic Hamiltonian. Equivalence refers to the principle that the two representations should yield identical observables such as energy eigenvalues.

The solution of the nuclear motion Schrödinger equation should not depend on whether the adiabatic or diabatic representations of the electronic states are used.^37^ In practice with numerical applications, observables should converge to the same values with increasing accuracy of calculation, e.g., by using increasingly larger basis sizes. Equivalency is often assumed but is rarely shown. Convergence between the adiabatic and diabatic states has been investigated in only a handful of papers. Zimmerman and George^38^ performed numerical convergence tests on adiabatic and diabatic states of the transition probability amplitudes in collisions of collinear atom–diatom systems, where the convergence to equivalence was demonstrated, and it was shown that convergence was markedly different with the diabatic representation converging significantly faster. Shi et al.^39^ evaluated numerical convergence rates of adiabatic and diabatic energy eigenvalues and eigenfunctions using a sinc-DVR method; equivalency was demonstrated, but this required using a complete adiabatic model and a conical intersection at high energy. The magnitude of the DDR corrections within the adiabatic representation has been studied before such as in the series of papers by Wolniewicz, Dressler, and co-workers,^40−46^ where excited electronic states of molecular hydrogen and their coupling were studied in detail. The earliest of these studies used the adiabatic approximation, but through the series, nonadiabatic couplings were introduced and improved for an increasing number of excited states and were shown to be essential to produce accurate spectroscopy (i.e., accurate rovibronic energies and transitions) of the system, as confirmed by comparison to experiment. In the later studies, the diabatic representation was also shown to provide an accurate description of the nuclear dynamics of H_2_, but comparisons between the adiabatic and diabatic representations were not shown. Additionally, DDR corrections were studied with respect to the computed rovibrational energies of H_2_^+^ and D_2_^+^ by Jaquet and Kutzelnigg^47^ and later by Jaquet^48^ on the H_2_^+^, H_3_^+^, and H_2_ systems. It is therefore expected that DDR contributions are critical for the accurate determination of the energies of small hydrogen-bearing molecules.

Nonadiabatic interactions are also important for scattering calculations, which often assume the equivalence between the adiabatic and diabatic representations.^49^ For example, Little and Tennyson^50^ provide a partial diabatic representation for the electronic structure of N_2_, which was used within multichannel quantum defect theory calculations for the dissociative recombination of N_2_^+^,^51^ where *ab initio* cross sections were generated. It was shown by Volkov et al.^52^ that for multichannel coulomb scattering calculations for the mutual neutralization reaction H^+^ + H^–^ → H_2_^*^→ H(1) + H(*n*), an adiabatic and diabatic reformulation produced not only equivalent results but also almost identical cross sections as generated from various other methods. Furthermore, the influence of the second derivative coupling term was shown to be important for producing accurate cross sections, an interesting result which showcases the need for accurate representation of nonadiabatic dynamics.

This study aims to show the numerical equivalence of the adiabatic and diabatic representations in nuclear motion calculations of rovibronic energies and spectral properties for two selected diatomic systems, represented by two coupled electronic states: yttrium oxide (YO) and carbon monohydride (CH) molecules illustrated in Figure 1. YO shows avoided crossings between the *B*^2^Σ^+^, *D*^2^Σ^+^ and *A*^2^Π, *C*^2^Π states as described by Yurchenko et al.^53^ YO has broad scientific interest, having been observed in stellar spectra,^54−57^ and found use in solar furnaces^58,59^ and magneto-optical traps.^60,61^ YO is a complex system showing many low-lying electronic states; accurate descriptions of its avoided crossings will be valuable to works in several fields. CH is one of the most studied free radicals^62^ because it occurs in such a wide variety of environments: it has been observed in flames,^63,64^ solar^65−67^ and stellar spectra,^68−70^ spectra of comets,^71^ ISM,^72−75^ and molecular clouds.^76^

As part of the study, we also report our implementation of the full diabatic/adiabatic treatments in our code Duo,^77^ a rovibronic solver of general coupled diatomic Schrödinger equations, which is used in the analyses. Duo is a general, open-access Fortran 2003 code (https://github.com/Exomol/Duo).

## 2 Description of the Diabatization of a Two-Electronic-State System

Consider a coupled two-electronic-state system of nuclear (pure vibrational) Schrödinger equations for a diatomic molecule in the adiabatic representation, with the nonadiabatic effects between these two states fully accounted for, as given by (ignoring spin and rotation angular momenta)
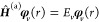
where *r* is the distance between the two nuclei and the Born–Huang 2 × 2 Hamiltonian operator is (see, e.g., Varga et al.,^30^ Römelt,^78^ and Yarkony et al.^79^)
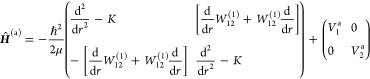
1Here, μ = *m*_1_*m*_2_/(*m*_1_ + *m*_2_) is the reduced mass, *V*_1_^(a)^(*r*) and *V*_2_^(a)^(*r*) are the adiabatic potential energy functions, and *W*_12_^(1)^(*r*) is the first-order DDR or nondiagonal NAC, given by
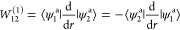
2where ψ_1_^a^ and ψ_2_^a^ are the adiabatic electronic wave functions, and *K*(*r*) is the diagonal DDR component given by
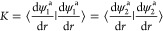
3Furthermore,  is the well-known DBOC.^80^

The derivative coupling *K*(*r*) is related to the second DDR *W*_12_^(2)^ through the following relations^81,82^ in the *g*-, *h*-, and *k*-notations
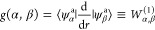
4
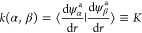
5
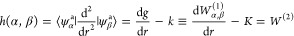
6In conjunction with eqs 4–6 and the results by Baer,^37^ Mabrouk and Berriche,^83^ and Smith,^84^ a simple and powerful expression for the matrix element of the diagonal DDR term *K* for the coupled two-electronic state problem is obtained
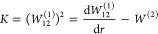
7A diabatic representation of a two-state system can be introduced via a unitary transformation ***U***(*r*) of the adiabatic electronic wave function vector , in which the first-order DDR vanishes and PECs and other molecular properties become smooth at the cost of introducing an off-diagonal potential energy coupling, termed a diabatic coupling (DC), between the nonadiabatically interacting electronic states.^17,18,85^ The unitary 2 × 2 matrix ***U***(*r*) is given by
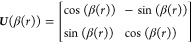
8where the mixing angle β(*r*) is obtained by integrating NAC as follows^8,86−88^
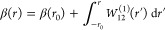
9where *r*_0_ is a reference geometry and is usually chosen as such that one can define a physical condition which ensures the mixing angle to equal π/4 at the crossing point *r*_c_. It can also be shown that for the diatomic one-dimensional case, the transformation to a strict diabatic basis is unique and that *W*_12_^(1)^ vanishes upon the diabatization together with *K*(*r*) (see eq 7). Similar to the work by Köppel et al.,^89^ who developed a Hamiltonian for the two-coupled electronic state problem, we develop theory for the diabatic and adiabatic electronic PECs for the coupled two-electronic states in question. The corresponding two-electronic-state Born–Huang Hamiltonian operator  then becomes
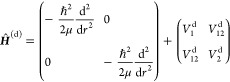
10where the diabatic potential energy functions *V*_1_^d^(*r*) and *V*_2_^d^(*r*) and the DC function *V*_12_^d^(*r*) are given by
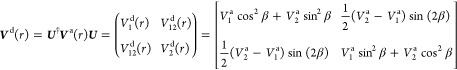
11

The goal of this work is to demonstrate the equivalency of the adiabatic and diabatic representations when solving the nuclear motion diatomic (eigenvalue) problem. To this end, we aim to construct, solve, and compare the eigensolutions of model diatomic systems in the adiabatic and diabatic representations.

If the adiabatic representation of an isolated two-electronic state diatomic system is fully defined by the three functions *V*_1_^a^(*r*), *V*_2_^a^(*r*), and *W*_12_^(1)^(*r*) in eq 1, in turn, the diabatic representation is fully defined by the three functions *V*_1_^d^(*r*), *V*_2_^d^(*r*), and *V*_12_^d^(*r*) in eq 10. In fact, both transformations can be fully described by a combination of any three functions from the set *V*_1_^a^(*r*), *V*_2_^a^(*r*), *W*_12_^(1)^(*r*), *V*_1_^d^(*r*), *V*_2_^d^(*r*), and *V*_12_^d^(*r*). For this study, we choose *V*_1_^d^(*r*), *V*_2_^d^(*r*), and *W*_12_^(1)^(*r*). The diabatic PECs *V*_1_^d^(*r*) and *V*_2_^d^(*r*) are expected to have smooth shapes by construction and are easy to parameterize, which explains our choice, while *W*_12_^(1)^(*r*) also has a rather simple, easy-to-parameterize cusp-like shape,^8,14,17−19^ as will be shown below. The other three functions are constructed from *V*_1_^d^(*r*), *V*_2_^d^(*r*), and *W*_12_^(1)^(*r*) as follows.

We first define β(*r*) via eq 9. By applying the inverse transformation ***U***^†^ to the potential matrix ***V***^d^(*r*) in eq 11, we arrive at the following condition for the off-diagonal element of the adiabatic potential matrix
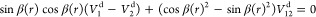
12which is required to be zero since ***V***^a^(*r*) = ***UV***^d^(*r*)***U***^**†**^ in eq 1 is diagonal by definition. Hence, we can rearrange it for the DC to get
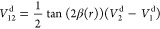
13

The adiabatic functions *V*_1_^a^(*r*) and *V*_2_^a^(*r*) can then be constructed as eigenvalues of the diabatic potential energy matrix (second term in eq 10)
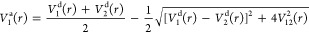
14
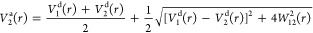
15or, equivalently, via the inverse unitary transformation ***U***
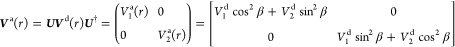
16

## 3 Spectroscopic Models

As an illustration, two model two-state electronic systems are used, YO and CH, with their diabatic and adiabatic curves shown in Figure 1 and introduced in detail in the following.

### 3.1 YO Spectroscopic Model

As an example of a two-state system with narrow, coupled-bound electronic curves, we chose the *ab initio* PEC curves of the *B*^2^Σ^+^ and *D*^2^Σ^+^ states of YO from Smirnov et al.^90^ with the NAC from Yurchenko et al.^53^

**Figure 2:**
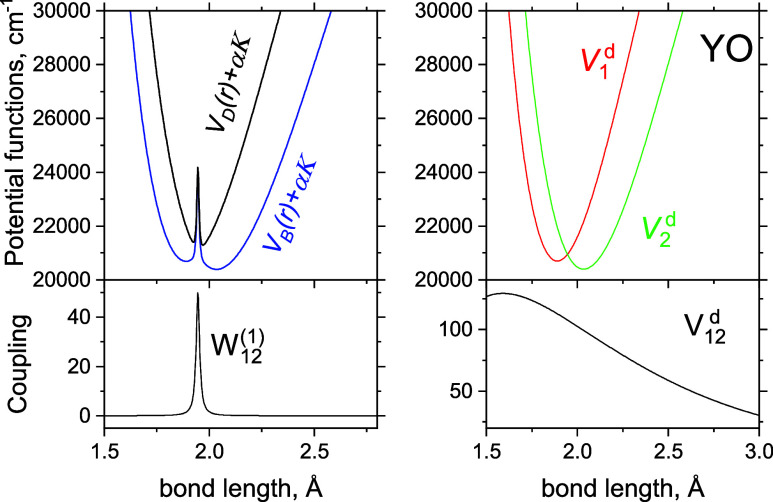
Full adiabatic (left) and diabatic (right) models of the *B*^2^Σ^+^ and *D*^2^Σ^+^ systems of YO. The top panels show the PECs, where the adiabatic PECs include the diagonal DDR corrections α*K* and α = *h*/(8π^2^*c*μ). The bottom panels show the corresponding coupling curves, NAC (left) and DC (right).

We use a Morse oscillator function as a simple model for the diabatic *B*^2^Σ^+^ and *D*^2^Σ^+^ PECs of YO as given by
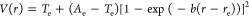
17where *A*_e_ is a dissociation asymptote, *A*_e_ – *V*(*r*_e_) is the dissociation energy, and *r*_e_ is an equilibrium distance of the PEC. The NAC of YO can be efficiently described by a Lorentzian function
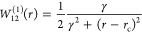
18where γ is the corresponding half-width-at-half-maximum (HWHM), while *r*_c_ is its center, corresponding to the crossing point of diabatic curves. These PECs and NACs are illustrated in Figure 2. The parameters defining these curves are listed in Table 1, which were obtained by fitting them to the corresponding *ab initio* data.

For the Lorentzian as a NAC, eq 9 is easily integrable to give the transformation angle β(*r*)
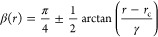
19where *r*_c_ is obtained as the crossing point between the PECs, and the ± sign refers to the path integral when *r* < *r*_c_ and *r*_c_ < *r*, respectively.

The adiabatic curves obtained using eqs 14 and 15 and the DC curve obtained using eq 13 are shown in Figure 2. The value of the crossing point *r*_c_ is obtained as a numerical solution of *V*_1_^d^ = *V*_2_^d^ and is listed in Table 1. The derivative coupling *K* in the diagonal matrix element of the adiabatic kinetic energy operator in eq 1 is simply defined by  according to eq 7. All of the corresponding curves are programmed in Duo analytically and are provided on a grid of 1000 equidistant bond lengths as part of the Supporting Information.

**Table 1:** Molecular Parameters Defining the YO Spectroscopic Model

parameter	*V*_1_^d^	*V*_2_^d^	*W*_12_^(1)^
*T*_e_, cm^–1^	20700.0	20400.0	
*r*_e_, Å	1.89	2.035	
*b*, Å^–^^1^	1.5	1.26	
*A*_e_, cm^–1^	59220.0	59220.0	
γ, cm^–^^1^			0.01
*r*_c_, Å			1.945843834

### 3.2 CH Spectroscopic Model

The spectroscopic model for CH, with curves illustrated in Figure 1 (right panel), is constructed to mimic the *ab initio* curves of *C*^1^Σ^+^ and 2^1^Σ^+^ by van Dishoeck.^91^ The *C*^1^Σ^+^ state has a bound shape with a well of about 16,700 cm^–1^ (2.0705 eV), which we model using a Morse oscillator function in eq 17. The 2^1^Σ^+^ state is repulsive, with the dissociation energy lower than that of *C*^1^Σ^+^ by about 10,000 cm^–1^. We chose to model the 2^1^Σ^+^ PEC using the following form
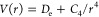
20The corresponding NAC between *C*^1^Σ^+^ and 2^1^Σ^+^ of CH from van Dishoeck^91^ is modeled using a two-parameter Lorentzian function in eq 18. All parameters defining the CH spectroscopic model are given in Table 2. As above, the value of the crossing point *r*_c_ is obtained as a numerical solution of *V*_1_^d^ = *V*_2_^d^.

**Table 2:** Molecular Parameters Defining the CH Diabatic Spectroscopic Model

parameter	*V*_1_^d^	*V*_2_^d^	*X*^1^Π	*W*_12_^(1)^
*T*_e_, cm^–1^	32500.0		0.0	
*r*_e_, Å	1.12		1.12	
*b*, Å^–^^1^	2.5		1.968	
*A*_e_, cm^–1^	49200.0	29374.0	39220.0	
*C*_4_, Å^–4^		18000.0		
γ, cm^–^^1^				0.2
*r*_c_, Å				1.656644935

## 4 Solving the Rovibronic Schrödinger Equations for CH and YO

Both CH and YO doublet systems represent open-shell molecules. Toward a complete rovibronic solution, the pure vibrational Hamiltonian operator in eqs 1 or 10 is extended with the rotation-spin-electronic contribution as follows (see Yurchenko et al.^77^ for details of the approach used)
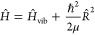
21where the rotational angular momentum operator *R̂* is replaced with
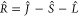
22Here, *Ĵ*, *Ŝ*, and *L̂* are the total, spin, and electronic angular momenta, respectively. We then solve the aforementioned rovibronic Schrödinger systems for YO and CH variationally on the Hund’s case (a) basis using the Duo program,^77^ which has been extended as part of this work to include the adiabatic and diabatic effects. The spectroscopic models of CH and YO are provided in the form of the Duo input files in both the diabatic and adiabatic representations as part of the Supporting Information.

Duo uses the numerical sinc-DVR method^92,93^ to solve the Schrödinger systems for the curves defined either on a grid or as analytic functions. For the analytic representations above, the corresponding functions are mapped on a grid of sinc-DVR points. For the grid input, cubic splines are used. The Duo kinetic energy has been extended to include the first derivative component required for implementation of the NAC, also using the sinc-DVR representation.^94^ The DBOC terms can be either provided as input or generated from the NAC using eq 3. In order to facilitate the numerically exact equivalency of the diabatic and adiabatic representations in Duo calculations, eqs 13–15 are provided and are used for constricting *V*_12_^d^, *V*_1_^a^(*r*), and *V*_2_^a^(*r*), respectively, from *V*_1_^d^(*r*), *V*_2_^d^(*r*), and β(*r*).

### 4.1 YO Solution

We first find the vibronic (*J* = 0.5) energies of the coupled *B*^2^Σ^+^ and *D*^2^Σ^+^ systems in the adiabatic and diabatic representations as accurately as possible in order to establish a baseline and also to demonstrate the equivalency of the two representations. Even though we know that the diabatic and adiabatic solutions should be equivalent (i.e., identical within the calculation error), this is always subject to the convergence or other numerical limitations. For example, Duo uses a PEC-adapted vibrational basis set constructed by solving the pure vibrational problem, which will be different depending on the representation, diabatic or adiabatic, and thus will influence the convergence. The corresponding YO model curves are shown in Figure 2, where DBOC coupling *K* is included in the adiabatic PECs for clarity. There is a striking difference between the two models, with a large spike in the middle of the adiabatic PECs, yet we expect them to give the same eigenvalues and eigenfunctions.

A selected set of rovibronic energy term values (*J* = 0.5) computed using the two methods is listed in Table 3. The energies are indeed identical (within 2.5 × 10^–5^ cm^–1^), but the approximate quantum state labels as assigned by Duo are very different. Duo assigns quantum labels via the largest contribution from the corresponding basis sets, which in both cases are very different and so are their state interpretations, in which case we compare states of matching energy enumerator *n*.

**Table 3:** Rovibronic (*J* = 0.5) Energy Term Values (cm^–1^) of the *B*^2^Σ^+^ (B) and *D*^2^Σ^+^ (D) Systems of YO Computed Using the Adiabatic and Diabatic Representations^a^

*n*	adiabatic					diabatic			
	*Ẽ*	*Ẽ*(DDRs = 0)	*Ẽ*(*K* = 0)	state	*v*	*Ẽ*	*Ẽ*(*V*_12_ = 0)	state	*v*
1	0.000000	0.000000	0.000000	B	0	0.000000	0.000000	D	0
2	344.431810	347.928597	191.831751	B	1	344.431809	351.249676	B	0
3	561.079914	690.986320	492.221984	B	2	561.079921	549.732652	D	1
4	1009.133229	967.537324	983.098980	B	3	1009.133232	1002.246089	B	1
5	1108.354299	1132.062465	1129.463766	D	0	1108.354283	1095.516787	D	2
6	1612.539760	1553.296745	1777.897073	B	4	1612.539736	1637.352406	D	3
7	1688.323434	1897.761066	1868.635701	B	5	1688.323453	1647.646531	B	2
8	2179.350796	2008.167697	2345.749886	D	1	2179.350783	2175.239507	D	4
9	2297.569318	2465.488852	2396.923772	B	6	2297.569321	2287.451003	B	3
10	2718.929830	2689.784491	2839.568147	B	7	2718.929830	2709.178092	D	5
11	2928.147305	2925.374682	3115.611400	D	2	2928.147294	2921.659505	B	4
12	3247.771603	3395.227251	3377.138924	B	8	3247.771603	3239.168161	D	6
13	3559.124439	3442.432354	3666.238711	D	3	3559.124429	3550.272037	B	5
14	3772.447582	3862.695406	3963.866748	B	9	3772.447578	3765.209712	D	7
15	4181.801597	4167.979957	4373.535285	D	4	4181.801594	4173.288598	B	6
16	4295.897860	4333.054560	4472.298326	B	10	4295.897854	4287.302747	D	8
17	4783.958004	4805.617146	4913.118506	B	11	4783.958001	4790.709188	B	7
18	4829.238038	4866.961640	4961.045768	D	5	4829.238030	4805.447266	D	9
19	5320.626170	5275.859430	5497.071432	B	12	5320.626156	5319.643267	D	10
20	5417.844769	5552.275088	5610.459386	D	6	5417.844772	5402.533809	B	8

a The energies are listed relative to the lowest *J* = 0.5 state

Having established the numerical equivalence, we can now investigate the importance of different nonadiabatic couplings for the YO model. Three approximations are considered here: (A1) in the adiabatic model, both DDR terms are switched off (*W*_12_^(1)^ = *K* = 0); (A2) in the adiabatic model, the diagonal DDR is switched off (*K* = 0), but the NAC is included; and (A3) in the diabatic model, the diabatic coupling is set to zero (*V*_12_ = 0). The effects of these approximations on the calculated energies of YO (*J* = 0.5) are also shown in Table 3. For the adiabatic model, the omission of *K* (A2) has the overall largest impact, especially on the *B*^2^Σ^+^ term values. The omission of *V*_12_ from the diabatic model (A3) appears to be less damaging than the other two approximations. It is clear, however, that any degradation of theory leads to large errors, which is unacceptable for high-resolution applications. This is, in fact, the main conclusion of this work: the impact of dropping any nonadiabatic corrections from the model describing a system with crossings has to always be investigated.

Out of the two representations, the adiabatic model is usually considered to be more complex to work with. Its curves have complex shapes with the model being very sensitive to the mutual consistency of the curves *V*_1_^a^, *V*_2_^a^, and *W*_12_^(1)^ around the crossing point. The disadvantage of the diabatic representation is that it does not come out as a solution of the (adiabatic) electronic structure calculations directly and needs to be constructed either through a diabatization approach^8,14,20−30^ or approximated.

### 4.2 Eigenfunctions and Reduced Density

It is instructive to compare the eigenfunctions φ_*i*_^*J*,τ^(*r*) of the adiabatic and diabatic solutions and different approximations. To this end, we form reduced radial densities of the eigenstate in question. The eigenfunctions φ_*i*_^*J*,τ^ utilized by Duo are expanded in the basis set |*n*⟩
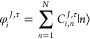
23where *N* is the basis size and *C*_*i*,*n*_^*J*,τ^ are the expansion coefficients used to assign quantum numbers by largest contributions. |*n*⟩ denotes the full basis: |*n*⟩ = |st, *J*, Ω, Λ, *S*, Σ, *v*⟩, where “st” is the electronic state; *S* is the electron spin angular momentum; *v* is the vibrational quantum number; and Λ, Σ, and Ω are the projections of electron orbital, spin, and total angular momentum along the internuclear axis, respectively. The reduced radial density ρ_*i*_^*J*,τ^(*r*) is then given by
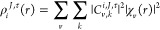
24where |*k*⟩ = |*st*, *J*, Ω, Λ, *S*, Σ⟩ and χ_*v*_(*r*) are the vibrational wave functions. The reduced density states are probability density functions over the bond length averaged over all quantum numbers in |*n*⟩. This is an efficient way of examining the behavior of the wave functions without looking in a hyperdimensional space defined by quantum numbers |*n*⟩.

**Figure 3:**
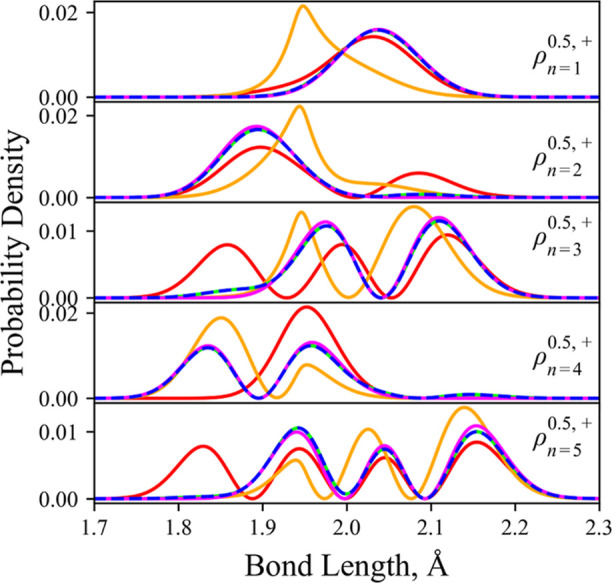
YO reduced density states for the lowest 5 bound levels with n being the energy enumerator given in Table 3. These reduced densities are illustrated and computed using different levels of theory: diabatic representation with DC (blue dotted); diabatic model with the DC turned off (magenta, A3); adiabatic representation with both the NAC and *K* correction included (lime green); adiabatic representation with NAC only (orange, A2); and adiabatic representation with no correction (red, A1).

Figure 3 shows selected reduced radial state densities of YO computed by using different representations and approximations. As expected from our energy comparisons, the diabatic and adiabatic representations produce identical results, whereas the reduced densities quickly deviate when the NAC and/or *K* corrections are removed. Again, it appears that the adiabatic representation with approximations is almost better when the DDRs are completely omitted rather than omitting only one, at least concerning the lower energy levels.

## 5 Adiabatic and Diabatic Solutions for CH

**Figure 4:**
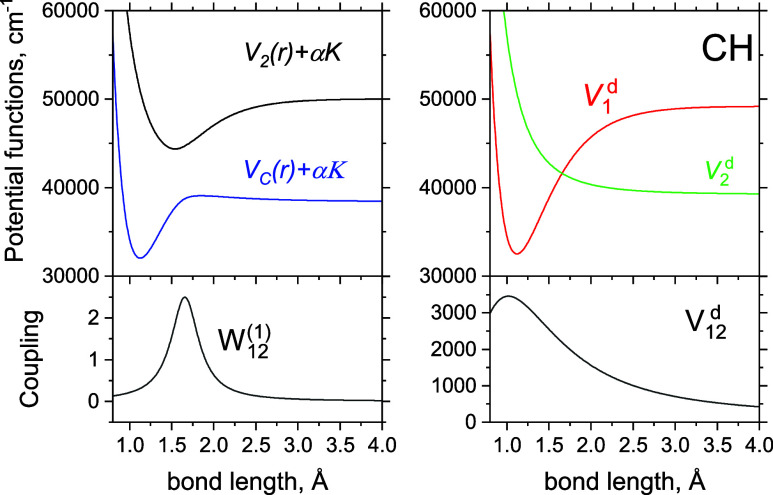
Full adiabatic (left) and diabatic (right) models of the *C*^1^Σ^+^ and 2^1^Σ^+^ systems of CH. The top panels show the PECs, where the adiabatic PECs include the diagonal DDR correction α*K*, where α = *h*/(8π^2^*c*μ). The bottom panels show the corresponding coupling curves, NAC (left) and DC (right).

We now turn to a slightly different system of the *C*^1^Σ^+^ and 2^1^Σ^+^ states of ^12^CH shown in Figure 4. Adiabatically, these states have a large separation and a broad NAC. In contrast to YO, there is no spike-type contribution from the DBOC-term *K* to the adiabatic PECs of CH. Diabatically, the system consists of a bound state and a repulsive state with a crossing at a large distance and high energy, which therefore should not influence the lower rovibronic states of *C*^2^Π significantly. Regardless of the representation used, the region above the first dissociation channel (39220.0 cm^–1^) is heavily (pre) dissociative and should contain both (pre)dissociative and continuum states. Duo is capable of finding both bound and continuum eigensolutions. While the bound wave functions satisfy the standard boundary condition leading to decay at large and short distances, the continuum wave functions can also be computed with the sinc-DVR method used by Duo and satisfy the boundary condition of vanishing exactly at the simulation box borders (together with their first derivatives), see Pezzella et al.^95^ For the analysis, we separate the (quasi-)bound and the continuum states by checking the character of the wave functions at the “right” border *r*_max_, while the continuum states tend to oscillate at *r* → ∞ with a nonzero density around *r*_max_(96) where the bound state vanishes completely.

The resulting energy term values of the bound states are listed in Table 4 for all five cases, including nonadiabatic and diabatic couplings considered as in the YO example. The full diabatic and adiabatic (bound) *C*^1^Σ^+^ energies are fully equivalent within 10^–6^ cm^–1^ (here shown up to the second decimal point). However, any degradation of the theory leads to drastic changes in the topology of the system and hence in the calculated rovibronic energies of the *C*^1^Σ^+^ state, with the accuracy quickly deteriorating already for *v* = 2. For example, by removing the DC term, the diabatic solution becomes meaningless with lots of nonphysically bound states above the first dissociation channel, nonexistent in the case of the full treatment. A similar effect is caused by the omission of the derivative couplings from the adiabatic pictures with bound spurious 2^1^Σ^+^ states produced by the adiabatically bound PEC 2^1^Σ^+^ (see Figure 4). Although the omission of the *K*(*r*) term from the adiabatic solution seems harmless for the topology of the corresponding PECs, even this case leads to a spurious vibrational 2^1^Σ^+^ (*v* = 0) state. Therefore, the conclusion is that every nonadiabatic term should be considered important, unless proven otherwise.

**Table 4:** Rovibronic (*J* = 0.5, 1.5, and 2.5) Bound Energy Term Values (cm^–1^) of the *C*^1^Σ^+^ (C) and 2^1^Σ^+^ (2) Systems of CH Computed Using the Adiabatic and Diabatic Representations^a^

*J*	*e*/*f*	adiabatic					diabatic			
		state	*v*	*Ẽ*	*Ẽ*(DDRs = 0)	*Ẽ*(*K* = 0)	state	*v*	*Ẽ*	*Ẽ*(*V*_12_ = 0)
0.5	e	C	0	0.00	0.00	0.00	C	0	0.00	0.00
0.5	e	C	1	2450.23	2448.12	2446.42	C	1	2450.23	2524.70
0.5	e	C	2	4617.30	4608.42	4601.48	C	2	4617.30	4822.76
0.5	e	2	0		11191.50	13607.15	C	3		6894.18
0.5	e	2	1		12464.33		C	4		8738.95
0.5	e	2	2		13549.98		C	5		10357.08
0.5	e	2	3		14449.75		C	6		11748.57
0.5	e	2	3				C	7		12913.41
0.5	e	2	3				C	8		13851.60
0.5	e	2	3				C	9		14563.15
0.5	f	2	2	27.83	27.82	27.82	C	6	27.83	28.08
0.5	f	2	3	2476.23	2474.10	2472.39	C	7	2476.23	2551.11
0.5	f	C	0	4641.14	4632.21	4625.24	C	8	4641.14	4847.45
0.5	f	2	1		11205.63	13620.21	C	9		6917.10
0.5	f	2	2		12478.11		C	0		8760.06
0.5	f	2	3		13562.86		C	1		10376.30
0.5	f	2	4		14461.35		C	2		11765.83
0.5	f	2	4				C	3		12928.61
0.5	f	2	4				C	4		13864.61
0.5	f	2	4				C	5		14573.78

a The energies are listed relative to the lowest *J* = 0.5 state

**Figure 5:**
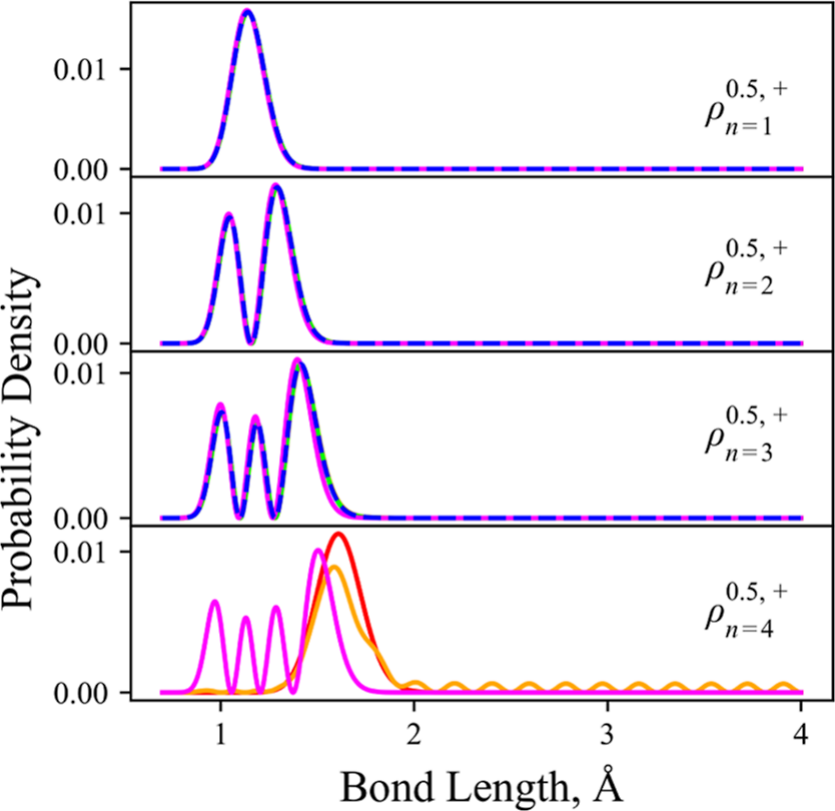
CH reduced density states for the lowest four bound rovibronic levels with *n* being the energy enumerator given by the row number in Table 4. Different levels of theory are used to compute these reduced densities and are illustrated: diabatic representation with DC (blue dotted); diabatic model with the DC turned off (magenta, A3); adiabatic representation with both the NAC and *K*(*r*) correction included (lime green); adiabatic representation with NAC only (orange, A2); and adiabatic representation with no correction (red, A1).

The corresponding reduced densities for some lower lying bound states of CH (*C*^1^Σ^+^, *J* = 0.5) are shown in Figure 5 (*n* = 1, 2, 3). We see that the low-lying vibronic states of *C*^2^Π are largely unaffected by the omission of the DDRs or DCs since they are energetically well separated from the region of nonadiabatic interaction, in this case occurring near dissociation. However, the reduced densities of the 2^1^Σ^+^ state (*n* = 4) quickly diverge when the NAC and/or *K* corrections are removed. The 2^1^Σ^+^ state is adiabatically bound and diabatically unbound, where this drastic difference is seen with the reduced densities in Figure 5 and corresponds to energy levels that arise from PECs of very different character. For example, in the diabatic case where the DC is omitted, the *n* = 4 state corresponds to the bound *C*^1^Σ^+^ (*J* = 0.5, +, *v* = 3) state, whereas in the adiabatic A1 and A2 cases, the *n* = 4 bound state corresponds to the bound 2^1^Σ^+^ (0.5, +, *v* = 0) state. In the cases where the DDRs and DCs are fully accounted for, no fourth bound state exists since the couplings will push it into the quasi-bound region about the adiabatic potential hump of the *C*^1^Σ^+^ state. This quasi-bound nature begins to show itself in the reduced density of the adiabatic case with *K* = 0, where small oscillations propagating to the right simulation border at 4 Å are seen.

### 5.1 Continuum Solution of CH: Photoabsorption Spectra

**Figure 6:**
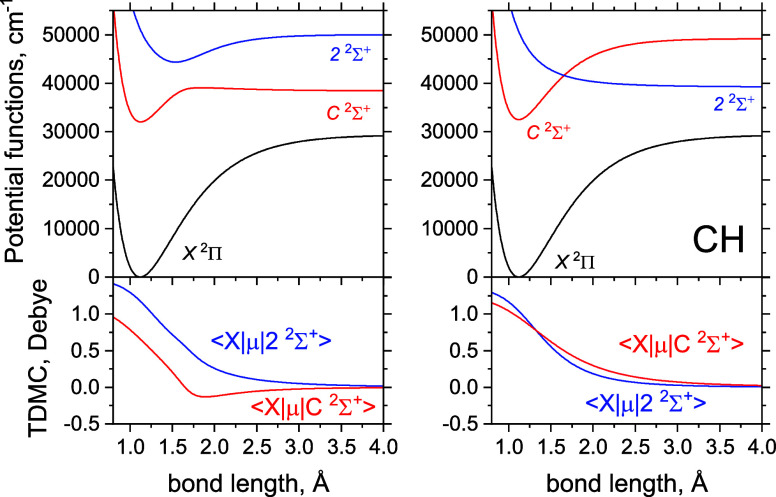
Adiabatic (left) and diabatic (right) models of the photoabsorption system of *X*^1^Π → *C*^1^Σ^+^/2^1^Σ^+^ of CH. The top panels show the PECs, adiabatic and diabatic, while the bottom panels show the corresponding TDM curves.

In order to illustrate the equivalence of the continuum solution involving the repulsive 2^1^Σ^+^ state of CH, we model a photoabsorption spectrum *X*^1^Π → *C*^1^Σ^+^/2^1^Σ^+^, where we follow the recipe from Pezzella et al.^97^ and Tennyson et al.^98^ For the *X*^1^Π state, we use the same Morse function representation in eq 17 with the parameters listed in Table 2. For the transition electric dipole moments  = ⟨*X*^1^Π|μ|*C*^1^Σ^+^⟩ and  = ⟨*X*^1^Π|μ|2^1^Σ^+^⟩ of CH, we adopt the *ab initio* curves by van Dishoeck^91^ with an approximate model using the following function
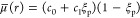
25where ξ_p_ is the Šurkus^99^ variable given by
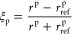
26The parameters defining the diabatic transition dipole moment (TDM) functions are listed in Table 5. The adiabatic TDM curves are obtained through the unitary transformation *U*(*r*)
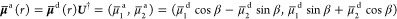
27where β(*r*) is from eq 9 and  and  are the diabatic TDM curves of ⟨*X*^1^Π|μ|*C*^1^Σ^+^⟩ and ⟨*X*^1^Π|μ|2^1^Σ^+^⟩, respectively. The full photodissociation system, in both adiabatic and diabatic representations, is illustrated in Figure 6.

**Table 5:** Molecular Parameters Defining the CH Diabatic Transition Dipole Moment Functions

parameter	⟨*X*^1^Π|μ|*C*^1^Σ^+^⟩	⟨*X*^1^Π|μ|2^1^Σ^+^⟩
*r*_ref_, Å	1.4	1.27
*P*	4	5
*c*_0_, Debye	0.71	0.85
*c*_1_, Debye	0.09	0.17

**Figure 7:**
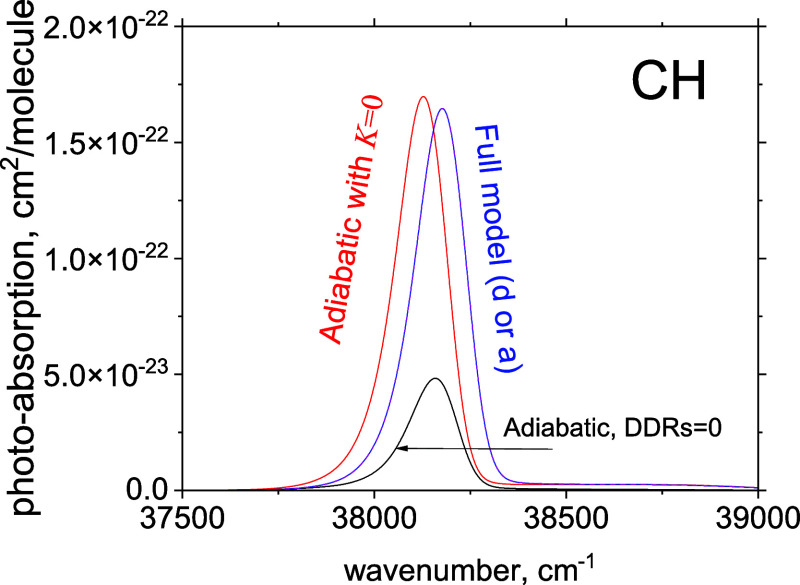
Photoabsorption spectra of CH at *T* = 300 K. The no-approximation case is shown with the blue line; the NAC = 0 case is shown with the red line; and the black line shows the spectrum with all DDRS set to zero.

**Figure 8:**
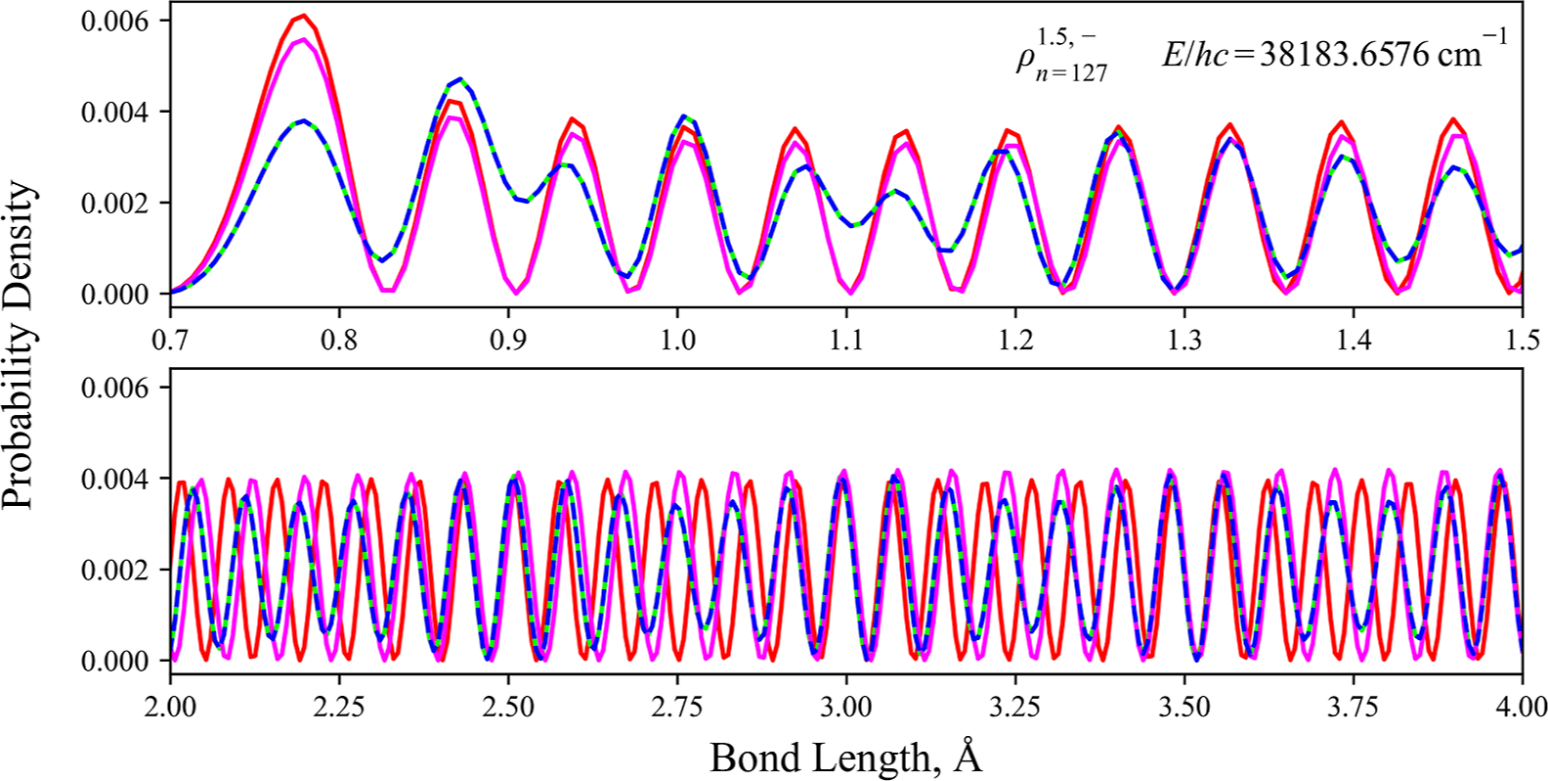
Reduced density of the continuum state corresponding to an energy of *hc*·38183.6576 cm^–1^. Its transition with the *X*^1^Π (*J* = 1.5, *f*, *v* = 0) state is positioned at the peak in the spectra of Figure 7. The reduced density state is illustrated and computed using different levels of theory: diabatic representation with DC (blue dotted); diabatic model with the DC turned off (magenta, A3); adiabatic representation with both the NAC and *K* correction included (lime green); adiabatic representation with NAC only (orange, A2); and adiabatic representation with no correction (red, A1).

Figure 7 shows a photoabsorption spectrum of CH at *T* = 300 K computed with Duo using the continuum solution of the coupled *C*^1^Σ^+^/2^1^Σ^+^ system from the bound states of *X*^1^Π for the diabatic and adiabatic models. We used the box of 60 Å and 1600 sinc-DVR points. For the cross sections, a Gaussian line profile of the HWHM of 50 cm^–1^ was used to redistribute the absorption intensities between the “discrete” lines representing the photoabsorption continuum. For details, see Pezzella et al.^97^ The diabatic and adiabatic continuum wave functions are obtained identically, so the photoabsorption spectra in this figure are indistinguishable. Figure 7 also illustrates the effects of the nonadiabatic approximations on the photoabsorption spectra of CH. Removing the diagonal DDR (*K* = 0) results in a shift of the band by about −50 cm^–1^, while setting both DDRs to zero leads to a significant drop of the absorption by a factor of ∼4. If we remove the DC term from the diabatic model, the bound absorption becomes dominant in the Franck–Condon region (see Figure 6), and the photoabsorption contribution drops by 2 orders of magnitude and is therefore not visible on this scale. As a further illustration of the continuum system of CH, Figure 8 gives an example of reduced densities of one of the continuum states used in the photoabsorption simulations.

## 6 Convergence

Since Duo uses a solution of the *J* = 0 uncoupled vibrational problem to form its vibrational PEC-optimized basis set functions ψ_*v*_(*r*), and these model problems are hugely different depending on the representation, one can also expect the convergence of the eigensolution to be impacted by the choice of the representation.

**Figure 9:**
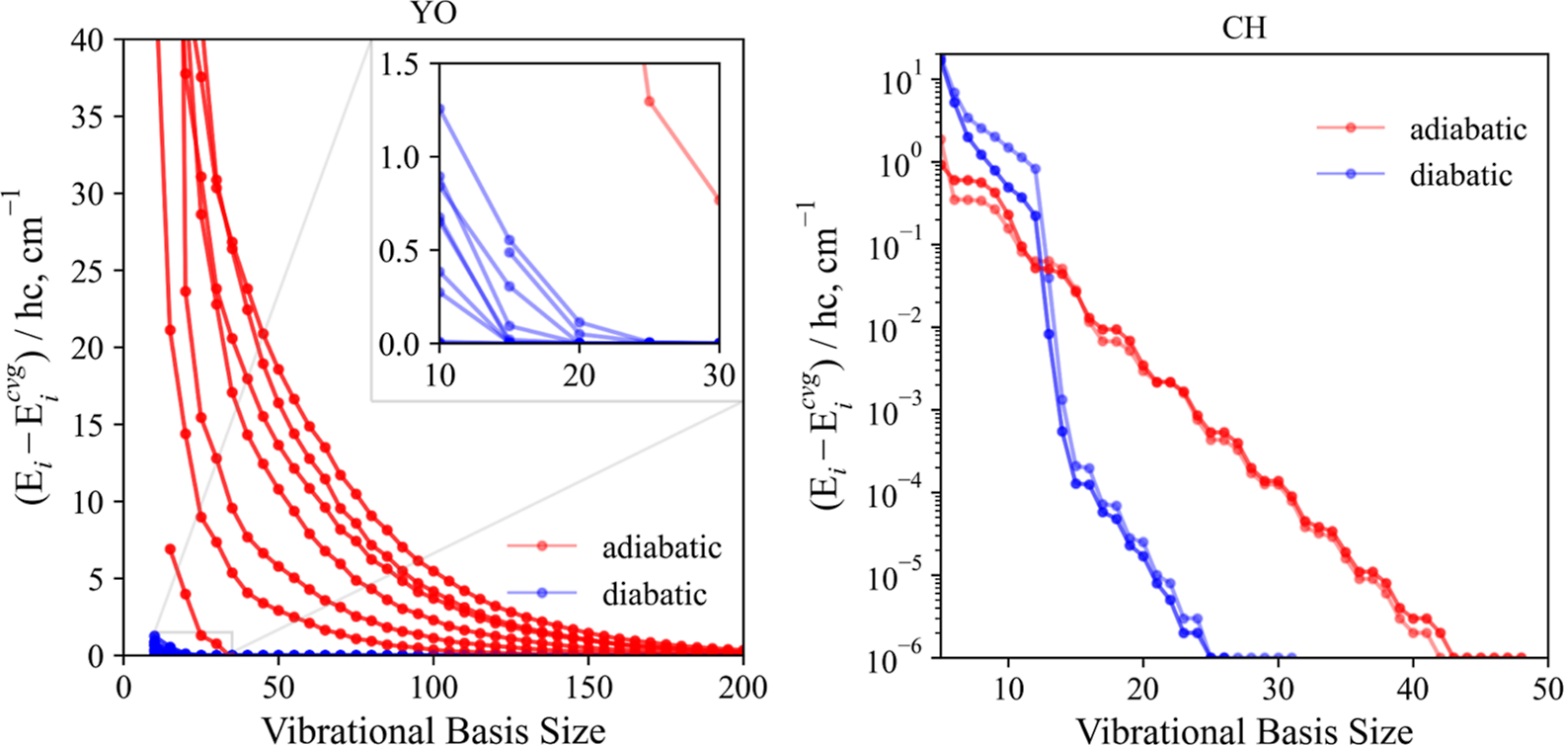
Convergence of the lowest 20 vibrational *J* = 0 energies of the *D*^2^Σ^+^ state of YO (left) and the C ^2^Σ^+^ (*v* = 0, *e*/*f*) state of CH (right) is plotted, where the difference of the *i*-th vibrational level *E*_*i*_ to its converged value *E*_*i*_^cvg^ is plotted as a function of vibrational basis size. A constant grid size of *G* = 3001, 4001 points for the sinc-DVR basis set was used for the YO and CH states, respectively. We see that the diabatically computed energies for YO converge much faster than the adiabatic ones, whereas for CH, the opposite is true.

Here, we test the convergence of the *J* = 0.5 energy levels of our simplified YO and CH models in the diabatic and adiabatic representations where all nonadiabatic effects are encountered. Figure 9 illustrates the convergence of the lowest 20 *J* = 0.5 energies of YO and the *n* = 5 state of CH (*C*^2^Σ^+^(*J* = 0.5, ±)), where the difference of the *i*-th level  to its converged value  is plotted as a function of vibrational basis size. The two systems show contrasting results. The diabatically computed YO (*D*^2^Σ^+^) energies converge very quickly for basis sizes of ∼25, whereas, within the adiabatic representation, a much larger basis set of ∼250 was required to achieve convergence. For CH (*C*^2^Σ^+^), the adiabatic energies initially converge faster, but the diabatic energies eventually converge to within 10^–6^ cm^–1^ for a basis size of ∼25 as opposed to ∼42 for the adiabatic energies.

Tests comparing the convergence rates for vibrational energies of higher *J* resulted in the same conclusions as those above for the *J* = 0.5 case.

This shows that there is not one representation that rules over the other; it depends on the character of the avoided crossing, specifically in its position, the shape of the potentials approaching the crossing, and the separation of the adiabatic PECs. It is therefore important to consider the system of study before choosing a representation where all corrections must be included.

## 7 Conclusions

A demonstration of the equivalency of the diabatic and adiabatic representations for two model diatomic systems, bound electronic *B*^2^Σ^+^ and *D*^2^Σ^+^ states of YO and a bound/repulsive electronic systems *C*^1^Σ^+^ and 2^1^Σ^+^ of CH, is presented. Both representations should be equivalent by construction, but we explicitly show this within nuclear motion calculations through comparison of the rovibronic energies and wave functions. The importance of different nonadiabatic couplings in the molecular Hamiltonian is investigated, such as how the rovibronic energies and wave functions change when the NAC, DBOC, or the diabatic coupling vanish.

We present a transformation from the adiabatic to strict diabatic basis for an isolated two-electronic state diatomic system. Each representation is defined by three functions; the adiabatic representation is given by two avoiding PECs and their corresponding NAC, whereas the diabatic picture is analogously defined by two diabatic PECs and a DC, all of which are related to each other through the mixing angle. Because of this, any three of the aforementioned quantities can be used to fully reconstruct either the adiabatic or the diabatic representation. We demonstrate that the choice of two diabatic PECs and a NAC provides an easily parameterizable and powerful way to define the two-level problem. In the case of the diabatic PECs, they can be modeled easily by Morse oscillators, and the NAC is easily modeled using a Lorentzian.

We show that omission of any of the nonadiabatic terms leads to significant changes in the spectral properties of these systems, which is unsatisfactory, especially for high-resolution applications. Even the diagonal derivative coupling, often omitted in practical applications, is shown to be of central importance in achieving equivalency.

We also show that the choice of a preferable representation, diabatic or adiabatic, is not the same for all systems. For cases where the NAC is small (large DC), then the adiabatic representation shows initially fast convergence of rovibronic energy levels. However, for cases where the NAC is large (small DC), the diabatic representation converges rovibronic energies with very small basis sets, where large ones are required for the corresponding adiabatic representation.

We used simplified approximated functions to model different diabatic and adiabatic curves for the purpose of facilitating the comparison and demonstration of equivalency as well as simplifying the debugging process. In fact, our program Duo uses numerically defined curves either provided as grids of *r*-dependent values or generated from analytic input functions, as used here. For the convenience of the reader, all curves from this work are provided in both the analytic and numerical representations as ASCII files, which are also Duo input files. As we demonstrated, the models provide the exact equivalency of the diabatic and adiabatic solutions and therefore can be used as a benchmark for similar programs. At the same time, Duo provides an efficient platform to test different aspects of diabatizations in diatomic calculations, including the testing of different approximations. Duo is open-access, with an extended online manual and many examples.

It would be interesting to develop and apply a similar methodology for polyatomic molecules, where the derivative couplings cannot be fully transformed away. The exact equivalence of the two representations should still be possible to demonstrate numerically, even for a quasi-diabatic transformation. This work on triatomic molecules is currently underway. In the present diatomic study, we show that exclusion of the DDR couplings can lead to differences on the order of magnitude of 10s–100s of cm^–1^ in the energy wavenumbers, reinforcing the need for a careful error budget of all the approximations made when using them in high-resolution spectroscopic applications.

All of the DDR, potential energy, and DC curves are programmed in Duo analytically and are provided on a grid of 1000 equidistant bond lengths as part of the Supporting Information. The spectroscopic models of CH and YO are also provided in the form of Duo input files in both the diabatic and adiabatic representations as part of the Supporting Information.

## Supporting Information Available

Molecular system descriptions (PDF)
Spectroscopic models of the carbon mono-hydride (CH) and yittrium oxide (YO) molecules (ZIP)

## References

[ref1] Schuurman M. S.; Stolow A. Dynamics at Conical Intersections. Annu. Rev. Phys. Chem. 2018, 69, 427–450. 10.1146/annurev-physchem-052516-050721. 29490199

[ref2] Levine B. G.; Martínez T. J. Isomerization Through Conical Intersections. Annu. Rev. Phys. Chem. 2007, 58, 613–634. 10.1146/annurev.physchem.57.032905.104612. 17291184

[ref3] Whitlow J.; Jia Z.; Wang Y.; Fang C.; Kim J.; Brown K. R. Simulating conical intersections with trapped ions. arXiv 2023, arXiv:2211.07319 10.48550/arXiv.2211.07319. 37640856

[ref4] Jasper A. W.; Zhu C.; Nangia S.; Truhlar D. G. Introductory lecture: Nonadiabatic effects in chemical dynamics. Faraday Discuss. 2004, 127, 1–22. 10.1039/b405601a. 15471336

[ref5] Matsika S.; Krause P. Nonadiabatic Events and Conical Intersections. Annu. Rev. Phys. Chem. 2011, 62, 621–643. 10.1146/annurev-physchem-032210-103450. 21219147

[ref6] Yarkony D. R. Diabolical conical intersections. Rev. Mod. Phys. 1996, 68, 985–1013. 10.1103/RevModPhys.68.985.

[ref7] Shu Y.; Fales B. S.; Peng W.-T.; Levine B. G. Understanding nonradiative recombination through defect-induced conical intersections. J. Phys. Chem. Lett. 2017, 8, 4091–4099. 10.1021/acs.jpclett.7b01707. 28799771

[ref8] Karman T.; Besemer M.; van der Avoird A.; Groenenboom G. C. Diabatic states, nonadiabatic coupling, and the counterpoise procedure for weakly interacting open-shell molecules. J. Chem. Phys. 2018, 148, 094105 10.1063/1.5013091.

[ref9] Karman T.; van der Avoird A.; Groenenboom G. C. Communication: Multiple-property-based diabatization for open-shell van der Waals molecules. J. Chem. Phys. 2016, 144, 121101 10.1063/1.4944744. 27036418

[ref10] Ma Q.; Kłos J.; Alexander M. H.; van der Avoird A.; Dagdigian P. J. The interaction of OH (X ^2^Π) with H_2_: Ab initio potential energy surfaces and bound states. J. Chem. Phys. 2014, 141, 174309 10.1063/1.4900478. 25381516

[ref11] Kłos J.; Ma Q.; Alexander M. H.; Dagdigian P. J. The interaction of NO (X ^2^Π) with H_2_: Ab initio potential energy surfaces and bound states. J. Chem. Phys. 2017, 146, 114301 10.1063/1.4977992. 28330347

[ref12] de Jongh T.; Karman T.; Vogels S. N.; Besemer M.; Onvlee J.; Suits A. G.; Thompson J. O. F.; Groenenboom G. C.; van der Avoird A.; van de Meerakker S. Y. T. Imaging diffraction oscillations for inelastic collisions of NO radicals with He and D2. J. Chem. Phys. 2017, 147, 013918 10.1063/1.4981023. 28688409

[ref13] Kolbuszewski M.; Wright J. S.; Buenker R. J. Avoided crossings in potential curves of BF^2+^: A study of models for bonding in diatomic dications. J. Chem. Phys. 1995, 102, 7519–7529. 10.1063/1.469083.

[ref14] Werner H.; Meyer W. MCSCF study of the avoided curve crossing of the two lowest ^1^Σ^+^ states of LiF. J. Chem. Phys. 1981, 74, 5802–5807. 10.1063/1.440893.

[ref15] Šimsová-Zámecníková M.; Soldán P.; Gustafsson M. Formation of NaCl by radiative association in interstellar environments. Astron. Astrophys. 2022, 664, A5 10.1051/0004-6361/202142965.

[ref16] Neumann J. V.; Wigner E. Quantum Chemistry: Classic Scientific Papers; World Scientific, 2000; pp 25–31.

[ref17] Mead C. A.; Truhlar D. G. Conditions for the definition of a strictly diabatic electronic basis for molecular systems. J. Chem. Phys. 1982, 77, 6090–6098. 10.1063/1.443853.

[ref18] Jasper A. W.; Kendrick B. K.; Mead C. A.; Truhlar D. G. Modern Trends in Chemical Reaction Dynamics; World Scientific, 2004; pp 329–391.

[ref19] Shu Y.; Varga Z.; Sampaio de Oliveira-Filho A. G.; Truhlar D. G. Permutationally Restrained Diabatization by Machine Intelligence. J. Chem. Theory Comput. 2021, 17, 1106–1116. 10.1021/acs.jctc.0c01110. 33405927

[ref20] Brady R. P.; Yurchenko S. N.; Kim G.-S.; Somogyi W.; Tennyson J. An ab initio study of the rovibronic spectrum of sulphur monoxide (SO): diabatic vs. adiabatic representation. Phys. Chem. Chem. Phys. 2022, 24, 24076–24088. 10.1039/D2CP03051A. 36172791 PMC9623608

[ref21] Zhu X.; Yarkony D. R. On the Construction of Property Based Diabatizations: Diabolical Singular Points. J. Phys. Chem. A 2015, 119, 12383–12391. 10.1021/acs.jpca.5b07705. 26444643

[ref22] Hoyer C. E.; Xu X.; Ma D.; Gagliardi L.; Truhlar D. G. Diabatization based on the dipole and quadrupole: The DQ method. J. Chem. Phys. 2014, 141, 114104 10.1063/1.4894472. 25240342

[ref23] Ruedenberg K.; Atchity G. J. A quantum chemical determination of diabatic states. J. Phys. Chem. 1993, 99, 3799–3803. 10.1063/1.466125.

[ref24] Atchity G. J.; Ruedenberg K. Global potential energy surfaces for the lowest two 1 A′ states of ozone. Theoretical Chemistry Accounts: Theory, Computation, and Modeling (Theoretica Chimica Acta) 1997, 96, 176–194. 10.1007/s002140050220.

[ref25] Nakamura H.; Truhlar D. G. The direct calculation of diabatic states based on configurational uniformity. J. Chem. Phys. 2001, 115, 10353–10372. 10.1063/1.1412879.

[ref26] Nakamura H.; Truhlar D. G. Direct diabatization of electronic states by the fourfold way. II. Dynamical correlation and rearrangement processes. J. Chem. Phys. 2002, 117, 5576–5593. 10.1063/1.1500734.

[ref27] Nakamura H.; Truhlar D. G. Extension of the fourfold way for calculation of global diabatic potential energy surfaces of complex, multiarrangement, non-Born–Oppenheimer systems: Application to HNCO (S,S1). J. Chem. Phys. 2003, 118, 6816–6829. 10.1063/1.1540622.

[ref28] Xu X.; Yang K. R.; Truhlar D. G. Diabatic molecular orbitals, potential energies, and potential energy surface couplings by the 4-fold way for photodissociation of Phenol. J. Chem. Theory Comput. 2013, 9, 3612–3625. 10.1021/ct400447f. 26584115

[ref29] Subotnik J.; Yeganeh S.; Cave R.; Ratner M. Constructing diabatic states from adiabatic states: Extending generalized Mulliken-Hush to multiple charge centers with Boys localization. J. Chem. Phys. 2008, 129, 244101 10.1063/1.3042233. 19123489

[ref30] Varga Z.; Parker K. A.; Truhlar D. G. Direct diabatization based on nonadiabatic couplings: the N/D method. Phys. Chem. Chem. Phys. 2018, 20, 26643–26659. 10.1039/C8CP03410A. 30320314

[ref31] Werner H.-J.; Knowles P. J.; Knizia G.; Manby F. R.; Schütz M. Molpro: a general-purpose quantum chemistry program package. Wiley Interdiscip. Rev.: Comput. Mol. Sci. 2012, 2, 242–253. 10.1002/wcms.82.

[ref32] Mead C. A.; Truhlar D. G. Conditions for the definition of a strictly diabatic electronic basis for molecular systems. J. Chem. Phys. 1982, 77, 6090–6098. 10.1063/1.443853.

[ref33] Baer M. Integral equation approach to atom-diatom exchange processes. Phys. Rep. 1989, 178, 99–143. 10.1016/0370-1573(89)90137-3.

[ref34] Baer M. Topological effects in molecular systems: an attempt towards a complete theory. Chem. Phys. 2000, 259, 123–147. 10.1016/S0301-0104(00)00193-2.

[ref35] Baer M.; Alijah A. Quantized non-adiabatic coupling terms to ensure diabatic potentials. Chem. Phys. Lett. 2000, 319, 489–493. 10.1016/S0009-2614(00)00195-0.

[ref36] Baer M. Introduction to the theory of electronic non-adiabatic coupling terms in molecular systems. Phys. Rep. 2002, 358, 75–142. 10.1016/S0370-1573(01)00052-7.

[ref37] Baer M. Beyond Born-Oppenheimer: Electronic Nonadiabatic Coupling Terms and Conical Intersections; John Wiley & Sons, 2006.

[ref38] Zimmerman I. H.; George T. F. Numerical comparison between electronically adiabatic and diabatic representations for collinear atom-diatom collisions. J. Chem. Phys. 1975, 63, 2109–2114. 10.1063/1.431550.

[ref39] Shi H.-m.; Guo G.-h.; Sun Z.-g. Numerical convergence of the Sinc discrete variable representation for solving molecular vibrational states with a conical intersection in adiabatic representation. Chin. J. Chem. Phys. 2019, 32, 333–342. 10.1063/1674-0068/cjcp1812275.

[ref40] Wolniewicz L.; Dressler K. The EF and GK ^1^Σg^+^ states of hydrogen: Adiabatic calculation of vibronic states in H_2_, HD, and D_2_. J. Mol. Spectrosc. 1977, 67, 416–439. 10.1016/0022-2852(77)90050-9.

[ref41] Dressler K.; Gallusser R.; Quadrelli P.; Wolniewicz L. The EF and GK ^1^Σ_g_^+^states of hydrogen: Calculation of nonadiabatic coupling. J. Mol. Spectrosc. 1979, 75, 205–219. 10.1016/0022-2852(79)90117-6.

[ref42] Dressler K.; Wolniewicz L. Improved adiabatic corrections for the B ^1^Σ_u_^+^, C^1^Π_u_, and D^1^Π_u_ states of the hydrogen molecule and vibrational structures for H_2_, HD, and D_2_. J. Chem. Phys. 1986, 85, 2821–2830. 10.1063/1.451040.

[ref43] Quadrelli P.; Dressler K.; Wolniewicz L. Nonadiabatic coupling between the EF+GK+H ^1^Σ_g_^+^, I^1^Π_g_, and J^1^Δ_g_ states of the hydrogen molecule. Calculation of rovibronic structures in H_2_, HD, and D_2_. J. Chem. Phys. 1990, 92, 7461–7478. 10.1063/1.458181.

[ref44] Wolniewicz L.; Dressler K. Nonadiabatic energy corrections for the vibrational levels of the B and B′ ^1^Σ_u_^+^states of the H_2_ and D_2_ molecules. J. Chem. Phys. 1992, 96, 6053–6064. 10.1063/1.462647.

[ref45] Wolniewicz L.; Dressler K. Adiabatic potential curves and nonadiabatic coupling functions for the first five excited ^1^∑^+^_g_ states of the hydrogen molecule. J. Chem. Phys. 1994, 100, 444–451. 10.1063/1.466957.

[ref46] Yu S.; Dressler K. Calculation of rovibronic structures in the lowest nine excited ^1^Σ_g_^+^+^1^Π_g_+^1^Δ_g_ states of H_2_, D_2_, and T_2_. J. Chem. Phys. 1994, 101, 7692–7706. 10.1063/1.468263.

[ref47] Jaquet R.; Kutzelnigg W. Non-adiabatic theory in terms of a single potential energy surface. The vibration–rotation levels of and. Chem. Phys. 2008, 346, 69–76. 10.1016/j.chemphys.2008.02.068.

[ref48] Jaquet R. Investigation of the global second-derivative non-adiabatic contributions: Rovibrational energies of H_2_^+^, H_2_, and prospects for H_3_^+^ (Part II). J. Mol. Spectrosc. 2022, 384, 111585 10.1016/j.jms.2022.111585.

[ref49] Baer M. Adiabatic and diabatic representations for atom-molecule collisions: Treatment of the collinear arrangement. Chem. Phys. Lett. 1975, 35, 112–118. 10.1016/0009-2614(75)85599-0.

[ref50] Little D. A.; Tennyson J. An R-matrix study of singlet and triplet continuum states of N_2_. J. Phys. B: At. Mol. Opt. Phys. 2014, 47, 105204 10.1088/0953-4075/47/10/105204.

[ref51] Little D. A.; Chakrabarti K.; Mezei J. Z.; Schneider I. F.; Tennyson J. The dissociative recombination of N_2_^+^: an ab initio study. Phys. Rev. A 2014, 90, 052705 10.1103/physreva.90.052705.

[ref52] Volkov M. V.; Yakovlev S. L.; Yarevsky E. A.; Elander N. Adiabatic versus diabatic approach to multichannel Coulomb scattering for mutual neutralisation reaction H^+^ + H^–^ → H_2_^*^→H(1)+H(n). Chem. Phys. 2015, 462, 57–64. 10.1016/j.chemphys.2015.07.008.

[ref53] Yurchenko S. N.; Brady R. P.; Tennyson J.; Smirnov A. N.; Vasilyev O. A.; Solomonik V. G. ExoMol. line lists: Empirical Rovibronic spectra Yitrium Oxide (YO). Mon. Not. R. Astron. Soc. 2024, 527, 4899 10.1093/mnras/stad3225.

[ref54] Goranskii V. P.; Barsukova E. A. Comparative spectral analysis of the peculiar red novae V838 Mon and V4332 Sgr in quiescence after their outbursts. Astron. Rep. 2007, 51, 126–142. 10.1134/S1063772907020072.

[ref55] Kaminski T.; Schmidt M.; Tylenda R.; Konacki M.; Gromadzki M. KECK/HIRES spectroscopy of V838 monocerotis in October 2005. Astrophys. J. Suppl. 2009, 182, 33–50. 10.1088/0067-0049/182/1/33.

[ref56] Murty P. S. New identifications of YO and CeO in R-Cygni. Astrophys. Lett. 1982, 23, 7–9.

[ref57] Murty P. S. PI-gruis: Molecular identifications and spectral classification. Astrophys. Space Sci. 1983, 94, 295–305. 10.1007/BF00653719.

[ref58] Badie J. M.; Cassan L.; Granier B. Temperature of the gas phase in solar processes from simulation of the YO fluorescence spectra for A^2^Π_1/2_-X^2^Σ^+^, A^2^Π_3/2_-X^2^Σ^+^, B^2^Σ^+^-X^2^Σ^+^systems. Eur. Phys. J.-Appl. Phys. 2005, 32, 61–64. 10.1051/epjap:2005070.

[ref59] Badie J. M.; Cassan L.; Granier B.; Agudelo Florez S.; Chejne Janna F. Gas temperature measurements in high concentration solar furnace environments: Evidence of nonequilibrium effects. J. Sol. Energy Eng. Trans.-ASME 2007, 129, 412–415. 10.1115/1.2769718.

[ref60] Yeo M.; Hummon M. T.; Collopy A. L.; Yan B.; Hemmerling B.; Chae E.; Doyle J. M.; Ye J. Rotational state microwave mixing for laser cooling of complex diatomic molecules. Phys. Rev. Lett. 2015, 114, 223003 10.1103/PhysRevLett.114.223003. 26196620

[ref61] Collopy A. L.; Ding S.; Wu Y.; Finneran I. A.; Anderegg L.; Augenbraun B. L.; Doyle J. M.; Ye J. 3D Magneto-Optical Trap of Yttrium Monoxide. Phys. Rev. Lett. 2018, 121, 213201 10.1103/PhysRevLett.121.213201. 30517816

[ref62] Furtenbacher T.; Hegedus S. T.; Tennyson J.; Császár A. G. Analysis of the measured high-resolution doublet rovibronic spectra of ^12^CH and ^16^OH. Phys. Chem. Chem. Phys. 2022, 24, 19287–19301. 10.1039/D2CP02240K. 35929432 PMC9382695

[ref63] Williams S.; Green D. S.; Sethuraman S.; Zare R. N. Detection of trace species in hostile environments using degenerate four-wave mixing: methylidyne radical (CH) in an atmospheric-pressure flame. J. Am. Chem. Soc. 1992, 114, 9122–9130. 10.1021/ja00049a053.

[ref64] Versailles P.; Watson G. M.; Lipardi A. C.; Bergthorson J. M. Quantitative CH measurements in atmospheric-pressure, premixed flames of C_1_-C_4_ alkanes. Combust 2016, 165, 109–124. 10.1016/j.combustflame.2015.11.001.

[ref65] Lambert D. L. The abundances of the elements in the solar photosphere - VIII. Revised abundances of carbon, nitrogen and oxygen. Mon. Not. R. Astron. Soc. 1978, 182, 249–272. 10.1093/mnras/182.2.249.

[ref66] Mélen F.; Grevesse N.; Sauval A.; Farmer C.; Norton R.; Bredohl H.; Dubois I. A new analysis of the vibration-rotation spectrum of CH from solar spectra. J. Mol. Spectrosc. 1989, 134, 305–313. 10.1016/0022-2852(89)90317-2.

[ref67] Grevesse N.; Lambert D. L.; Sauval A. J.; van Dishoeck E. F.; Farmer C. B.; Norton R. H. Vibration-rotation bands of CH in the solar infrared spectrum and the solar carbon abundance. Astron. Astrophys. 1991, 242, 488–495.

[ref68] Ridgway S. T.; Carbon D. F.; Hall D. N. B.; Jewell J. An atlas of late-type stellar spectra, 2400–2778 inverse centimeters. Astrophys. J. Suppl. 1984, 54, 177–209. 10.1086/190925.

[ref69] Lambert D. L.; Gustafsson B.; Eriksson K.; Hinkle K. H. The Chemical Composition of Carbon Stars. I. Carbon, Nitrogen, and Oxygen in 30 Cool Carbon Stars in the Galactic Disk. Astrophys. J. Suppl. 1986, 62, 373 10.1086/191145.

[ref70] Masseron T.; Plez B.; Van Eck S.; Colin R.; Daoutidis I.; Godefroid M.; Coheur P.-F.; Bernath P.; Jorissen A.; Christlieb N. CH in stellar atmospheres: an extensive linelist. Astron. Astrophys. 2014, 571, A47 10.1051/0004-6361/201423956.

[ref71] Womack M.; Lutz B. L.; Wagner R. M. Pre- and postperihelon abundances of gas and dust in comet Halley. Astrophys. J. 1994, 433, 886 10.1086/174697.

[ref72] Swings P.; Rosenfeld L. Considerations Regarding Interstellar Molecules. Astrophys. J. 1937, 86, 483–486. 10.1086/143880.

[ref73] Jura M.; Meyer D. M. An optical measurement of the population inversion of the ground state Lambda doublet of interstellar CH. Astrophys. J. 1985, 294, 238–241. 10.1086/163292.

[ref74] Somerville W. B.; Crawford I. A. Observations of molecules in diffuse interstellar clouds. J. Chem. Soc. Faraday Trans. 1993, 89, 2261–2268. 10.1039/ft9938902261.

[ref75] Lien D. A reanalysis of the interstellar CH abundance. Astrophys. J. 1984, 284, 578–588. 10.1086/162440.

[ref76] Stacey G. J.; Lugten J. B.; Genzel R. Detection of Interstellar CH in the Far-Infrared. Astrophys. J. 1987, 313, 859 10.1086/165025.

[ref77] Yurchenko S. N.; Lodi L.; Tennyson J.; Stolyarov A. V. Duo: A general program for calculating spectra of diatomic molecules. Comput. Phys. Commun. 2016, 202, 262–275. 10.1016/j.cpc.2015.12.021.

[ref78] Römelt J. A Hermitean reformulation of the Born–Oppenheimer nonadiabatic coupling terms for diatomic molecules. Int. J. Quantum Chem. 1983, 24, 627–631. 10.1002/qua.560240609.

[ref79] Yarkony D. R.; Xie C.; Zhu X.; Wang Y.; Malbon C. L.; Guo H. Diabatic and adiabatic representations: Electronic structure caveats. Computational and Theoretical Chemistry 2019, 1152, 41–52. 10.1016/j.comptc.2019.01.020.

[ref80] Handy N. C.; Yamaguchi Y.; Schaefer H. F. The diagonal correction to the Born-Oppenheimer approximation — its effect on the singlet-tripet splitting of CH_2_ and other molecular effects. J. Chem. Phys. 1986, 84, 4481–4484. 10.1063/1.450020.

[ref81] Lengsfield B. H.; Yarkony D. R. On the evaluation of nonadiabatic coupling matrix elements for MCSCF/CI wave functions using analytic derivative methods. III. Second derivative terms. J. Chem. Phys. 1986, 84, 348–353. 10.1063/1.450144.

[ref82] Saxe P.; Yarkony D. R. On the evaluation of nonadiabatic coupling matrix elements for MCSCF/CI wave functions. IV. Second derivative terms using analytic gradient methods. J. Phys. Chem. 1987, 86, 321–328. 10.1063/1.452621.

[ref83] Mabrouk N.; Zrafi W.; Berriche H. Theoretical study of the LiNa molecule beyond the Born–Oppenheimer approximation: adiabatic and diabatic potential energy curves, radial coupling, adiabatic correction, dipole moments and vibrational levels. Mol. Phys. 2020, 118, e1605098 10.1080/00268976.2019.1605098.

[ref84] Smith F. T. Diabatic and Adiabatic Representations for Atomic Collision Problems. Phys. Rev. 1969, 179, 111–123. 10.1103/PhysRev.179.111.

[ref85] Delos J. B. Theory of electronic transitions in slow atomic collisions. Rev. Mod. Phys. 1981, 53, 287–357. 10.1103/RevModPhys.53.287.

[ref86] Simah D.; Hartke B.; Werner H.-J. Photodissociation dynamics of H2S on new coupled ab initio potential energy surfaces. J. Chem. Phys. 1999, 111, 4523–4534. 10.1063/1.479214.

[ref87] An H.; Baeck K. K. A practical and efficient diabatization that combines Lorentz and Laplace functions to approximate nonadiabatic coupling terms. J. Chem. Phys. 2015, 143, 194102 10.1063/1.4935607. 26590522

[ref88] Baeck K.; An H. Practical approximation of the non-adiabatic coupling terms for same-symmetry interstate crossings by using adiabatic potential energies only. J. Chem. Phys. 2017, 146, 064107 10.1063/1.4975323. 28201877

[ref89] Köppel H.; Domcke W.; Cederbaum L. S. Advances in Chemical Physics; John Wiley & Sons, Ltd, 1984; pp 59–246.

[ref90] Smirnov A. N.; Solomonik V. G.; Yurchenko S. N.; Tennyson J. Spectroscopy of YO from first principles. Phys. Chem. Chem. Phys. 2019, 21, 22794–22810. 10.1039/C9CP03208H. 31598617

[ref91] van Dishoeck E. F. Photodissociation processes in the CH molecule. J. Chem. Phys. 1987, 86, 196–214. 10.1063/1.452610.

[ref92] Guardiola R.; Ros J. On the numerical integration of the Schrödinger equation in the finite-difference schemes. J. Comput. Phys. 1982, 45, 374–389. 10.1016/0021-9991(82)90110-3.

[ref93] Lund J. R.; Riley B. V. A Sine-Collocation Method for the Computation of the Eigenvalues of the Radial Schrödinger Equation. IMA J. Numer. Anal. 1984, 4, 83–98. 10.1093/imanum/4.1.83.

[ref94] Lo J.; Shizgal B. D. Spectral convergence of the quadrature discretization method in the solution of the Schrödinger and Fokker-Planck equations: Comparison with sinc methods. J. Chem. Phys. 2006, 125, 194108 10.1063/1.2378622. 17129090

[ref95] Pezzella M.; Tennyson J.; Yurchenko S. N. ExoMol photodissociation cross-sections - I. HCl and HF. Mon. Not. R. Astron. Soc. 2022, 514, 4413–4425. 10.1093/mnras/stac1634.

[ref96] Yurchenko S. N.; Nogué E.; Azzam A. A. A.; Tennyson J. ExoMol line lists - XLVII. Rovibronic spectrum of aluminium monochloride (AlCl). Mon. Not. R. Astron. Soc. 2023, 520, 5183–5191. 10.1093/mnras/stac3757.

[ref97] Pezzella M.; Yurchenko S. N.; Tennyson J. A method for calculating temperature-dependent photodissociation cross sections and rates. Phys. Chem. Chem. Phys. 2021, 23, 16390–16400. 10.1039/D1CP02162A. 34318825 PMC8972202

[ref98] Tennyson J.; Pezzella M.; Zhang J.; Yurchenko S. N. Data structures for photoadsorption within the ExoMol project. RASTI 2023, 2, 231–237. 10.1093/rasti/rzad014.

[ref99] Šurkus A.; Rakauskas R. J.; Bolotin A. B. The Generalized Potential-Energy Function for Diatomic-Molecules. Chem. Phys. Lett. 1984, 105, 291–294. 10.1016/0009-2614(84)85032-0.

