# Supplementary material for: Numerical Equivalence of Diabatic and Adiabatic Representations in Diatomic Molecules
Source: J Chem Theory Comput. 2024 Jan 3;20(5):2127–39. doi: 10.1021/acs.jctc.3c01150 (PMC10938500; doi:10.1021/acs.jctc.3c01150)
Supplement: Supplementary file 1 — ct3c01150_si_001.pdf [file ct3c01150_si_001.pdf]

Supporting Information

The Numerical Equivalence of Diabatic and Adiabatic Representations in Diatomic Molecules

Ryan P. Brady, Charlie Drury, Sergei N. Yurchenko,* and Jonathan Tennyson

Department of Physics and Astronomy, University College London, Gower Street, WC1E 6BT London, United Kingdom

E-mail: s.yurchenko@ucl.ac.uk

The supplementary materials to this study contain the spectroscopic models of the open-shell carbon mono-hydride (CH) and yittrium oxide (YO) molecules in the form of DUO input files in both the adiabatic and diabatic representations; descriptions of these two systems are provided below. For each molecule, 10 input files are provided covering the cases of a full model and models using degraded theory (removing important coupling terms); an explanation of the different input files can be found below. With every DUO input file, a corresponding version where curves are defined on a grid are also provided.

SUPPLEMENTARY FILE DESCRIPTION

The supplementary files have the following naming convention:

`<molecule>_<representation>_<function-type>_<theory-level>.inp`,

where

- `<molecule>` : `CH` or `YO`

- `<representation>` : `ADIABATIC` or `DIABATIC`
- `<function-type>` : `analytic` or `'grid'\verb`. Analytic function type means the potentials and couplings are represented using analytic forms and are then mapped onto a grid of sinc-DVR points; grid means the curves are provided on a grid which are then interpolated using cubic-splines.
- `<theory-level>` : describes the level of theory used in the calculation. For the diabatic representation, `FULL-MODEL` means two PECs and their DC are provided; `DC=0` means the DC has been turned off in the calculation. For the adiabatic representation, `FULL-MODEL` means two avoided crossing PECs, a first-order NAC ($W^{(1)}$), and a DBOC ($K(r)$) coupling term are provided; `Keq0` ($K = 0$) means the DBOC $(r)K$ term has been turned off in the calculation; `W1eqKeq0` ($W^{(1)} = K = 0$) means that both the first-order NAC, $W^{(1)}(r)$, and the DBOC $K(r)$ coupling term have been turned off in the calculation. The suffix `_unbound` indicates the photo-absorption spectra.

MOLECULAR SYSTEM DESCRIPTIONS

YO: We consider the two strongly interacting lectronic states $B\,^2\Sigma^+$ and $D\,^2\Sigma^+$ which possess a large Non-Adiabatic-Coupling (NAC) and a weak Diabatic Coupling (`DC`, `diabatic` in the Duo input). They exhibit an avoided crossing near the potential minima and have strongly bound-characters.

CH: We consider the two weakly nteracting electronic states $C\,^1\Sigma^+$ and $2\,^1\Sigma^+$ which possess a weak NAC in the adiabatic representation and a strong DC in the diabatic representation. They exhibit an avoided crossing near the adiabatic dissociation limit of the C state and diabatically consist of a bound lower state and a repulsive/unbound upper state.
